# A multicenter paper-based and web-based system for collecting patient-reported outcome measures in patients undergoing local treatment for prostate cancer: first experiences

**DOI:** 10.1186/s41687-020-00224-7

**Published:** 2020-07-13

**Authors:** Christoph Kowalski, Rebecca Roth, Günther Carl, Günter Feick, Alisa Oesterle, Andreas Hinkel, Thomas Steiner, Marko Brock, Björn Kaftan, Rainer Borowitz, Niko Zantl, Axel Heidenreich, Andreas Neisius, Christopher Darr, Christian Bolenz, Burkhard Beyer, Jesco Pfitzenmaier, Bernhard Brehmer, Jan Fichtner, Björn Haben, Simone Wesselmann, Sebastian Dieng

**Affiliations:** 1 German Cancer Society, Kuno-Fischer-Strasse 8, 14057 Berlin, Germany; 2 Institute of Medical Statistics and Computational Biology, University of Cologne, Cologne, Germany; 3 Help for Prostate Cancer Patients (Förderverein Hilfe bei Prostatakrebs e.V., FHbP), Tornesch, Germany; 4 Federal Association of German Prostate Cancer Patient Support Groups, Bonn, Germany; 5 OnkoZert, Neu-Ulm, Germany; 6 Franziskus Hospital, Bielefeld, Germany; 7 Helios Klinikum Erfurt, Erfurt, Germany; 8 Marien Hospital Herne, Herne, Germany; 9 Städtisches Klinikum Lüneburg, Lüneburg, Germany; 10 Klinikum Memmingen, Memmingen, Germany; 11 Klinikum Singen, Singen, Germany; 12 Cologne University Hospital, Cologne, Germany; 13 Barmherzige Brüder Trier, Trier, Germany; 14 Essen University Hospital, Essen, Germany; 15 University of Ulm, Ulm, Germany; 16 Martini-Klinik Prostate Cancer Center Hamburg, Hamburg, Germany; 17 Evangelisches Klinikum Bethel Johannesstift, Bielefeld, Germany; 18 Diaconal Hospital of Schwäbisch Hall, Schwäbisch Hall, Germany; 19 Johanniter Krankenhaus Oberhausen, Oberhausen, Germany; 20 St. Marien Hospital Ahaus, Ahaus, Germany

**Keywords:** Multidisciplinary studies, Health services research, Prostate cancer, Patient-reported outcomes, Certification, Functional status, Improvement intervention, Implementation

## Abstract

**Purpose:**

To give an overview of the multicenter Prostate Cancer Outcomes (PCO) study, involving paper-based and web-based collection of patient-reported outcome measures (PROM) in patients undergoing local treatment for prostate cancer in certified centers in Germany. The PCO study is part of the larger Movember-funded TrueNTH Global Registry. The article reports on the study’s design and provides a brief progress report after the first 2 years of data collection.

**Methods:**

Prostate cancer centers (PCCs) certified according to German Cancer Society requirements were invited to participate in collecting patient-reported information on symptoms and function before and at least once (at 12 months) after treatment. The data were matched with disease and treatment information. This report describes progress in patient inclusion, response rate, and variations between centers relative to online/paper use, and also data quality, including recruitment variations relative to treatment in the first participating PCCs.

**Results:**

PCC participation increased over time; 44 centers had transferred data for 3094 patients at the time of this report. Patient recruitment varied widely across centers. Recruitment was highest among patients undergoing radical prostatectomy. The completeness of the data was good, except for comorbidity information.

**Conclusions:**

The PCO study benefits from a quality improvement system first established over 10 years ago, requiring collection and harmonization of a predefined clinical dataset across centers. Nevertheless, establishing a PROM routine requires substantial effort on the part of providers and constant monitoring in order to achieve high-quality data. The findings reported here may be useful for guiding implementation in similar initiatives.

## Introduction

The mortality rate associated with prostate cancer is low in comparison with other cancers, but patients may suffer from severe impairment of their functional status due to disease and treatment, particularly with regard to erectile function and continence [1]. There have been several reports well describing the benefits for cancer patients that result from monitoring their functional status and using functional status indicators for quality assurance and quality improvement [2–6]. However, such monitoring has not yet been widely implemented in routine care for prostate cancer. The German Cancer Society — together with the OnkoZert certification institute and the Federal Association of German Prostate Cancer Patient Support Groups and the Movember Foundation — has launched an outcomes data collection system that combines previously collected clinical data with a paper-based and web-based system for collecting patient-reported outcome measures (PROM) in routine prostate cancer care. This effort, known as the “Prostate Cancer Outcome (PCO)” study, is part of the larger TrueNTH Global Registry (TNGR) funded by the Movember Foundation [7].

The idea underlying the TNGR is to compare and reduce outcome variation (for the better) in relation to the positive deviance framework [8]. Briefly, this means identifying better-performing providers and “best practices” in order to help others learn from these. The data in the PCO study and the TNGR are collected in accordance with the International Consortium for Health Outcomes Measurement (ICHOM) standard set for localized prostate cancer [9], with a few additional items. All prostate cancer centers (PCCs) that are certified in accordance with the requirements of the DKG [10] were invited to participate. Patient recruitment began July 1, 2016. A German validation paper on the patient reported outcome instrument, the EPIC-26, which is in use in many large ongoing studies [11], was published recently [12]. The present article reports on the design of the PCO study and provides a brief progress report after the first 2 years of data collection.

## Materials and methods

### Brief study protocol

Since 2008, PCCs have been able to apply for certification in accordance with criteria set out by the German Cancer Society [10]. Certification requires centers to document clinical and treatment data that are used for annual audits. These data represent one of the two sources for patient data in the PCO study.

All PCCs were invited to participate in the PCO study in August 2015. Twenty-four centers expressed initial interest, while others still had the option to join later. During study preparation, infrastructure development, and PCC training, the study protocol was developed and approved by the ethics committee of the Medical Association of Berlin (Eth-12/16) in June 2016, with an amendment approved in June 2018.^1^ Patient recruitment started in July 2016. The study protocol was based on the TNGR protocol for collecting data in accordance with the ICHOM standard set, with few divergences reflecting specific aspects of certified centers and German legal matters, including an opt-in instead of an opt-out approach. The primary purposes of the study are to compare outcome variations (both patient-reported and clinical) and to make it possible to reduce differences (for the better) using individual symptom and function profiles. This includes identifying positively deviating providers and encouraging mutual learning. A further purpose is to evaluate whether the suggested data collection infrastructure works and to constantly improve it.

**Fig. 1:**
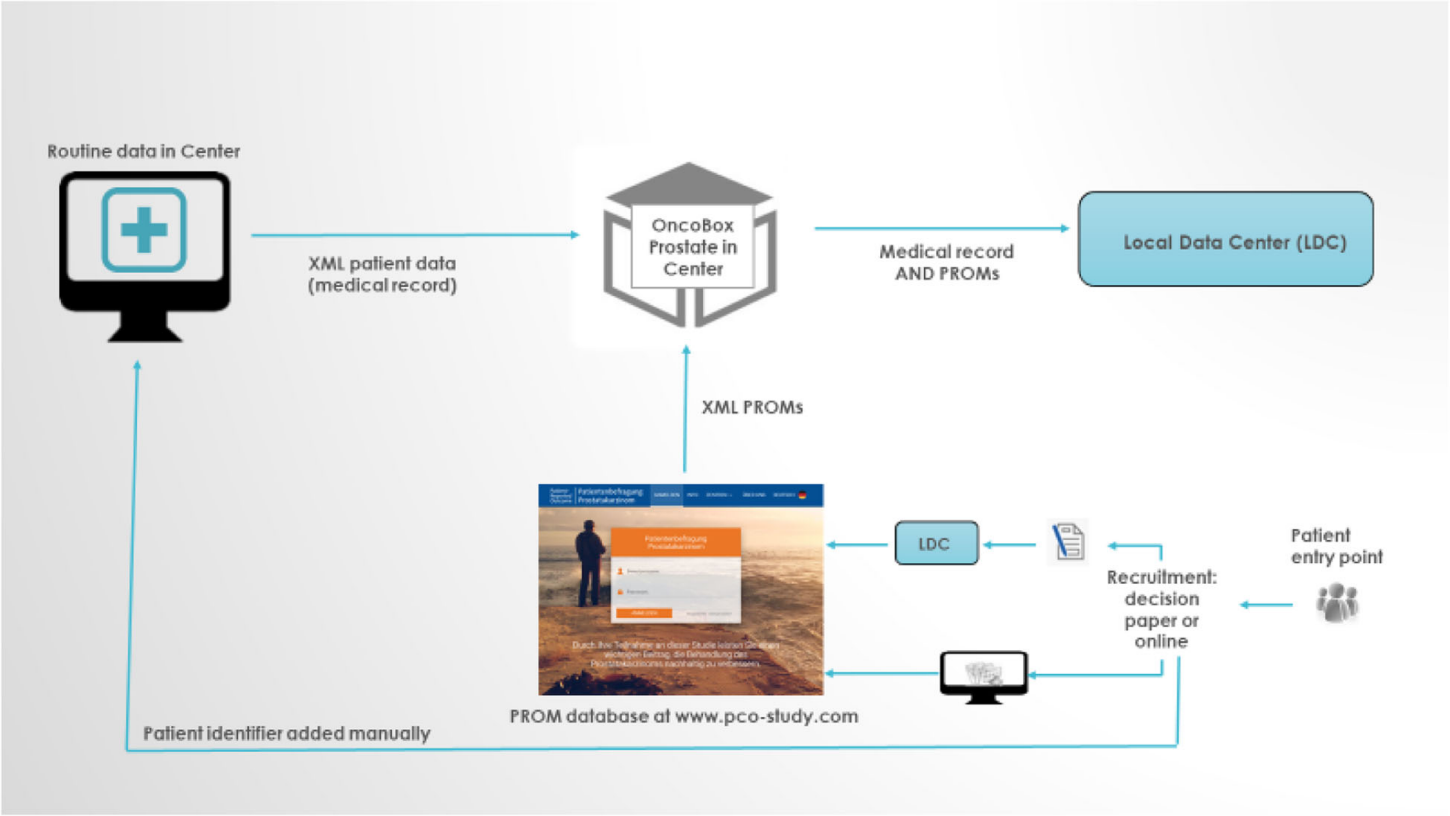
Data collecting infrastructure

Participating PCCs invite patients who are undergoing local treatment for prostate cancer — radiotherapy, radical prostatectomy including radical cystoprostatectomy —, as well as active surveillance or watchful waiting to give informed consent for the collection of data on patient, disease, and treatment characteristics needed for purposes of case-mix adjustment. These data include age, comorbidities, date of diagnosis, prostate-specific antigen (PSA) level, TNM categories, Gleason score, treatment choice (prior to recruitment and definitive), Clavien–Dindo grading, margin status etc., and were already collected as “routine data” (“routine data in center”, Fig. 1) in the center with all of them being used for other purposes as well except for comorbidities, which are documented exclusively for the study. In addition, the patients complete an initial questionnaire, which includes the EPIC-26 [13] to assess patient-reported symptoms and function before the start of definitive treatment as well as information on education, insurance, and citizenship not yet part of routine data. Patients and centers can decide whether they want questionnaires to be filled out paper-based or online. If patients complete the questionnaire on paper, it is sent to the local data center (LDC) by mail where questionnaires are entered into the PROM database manually and were scores are calculated. If patients fill out the online questionnaire, PROMs are entered in the database immediately. Upon data entry in the PROM database, the PCCs are notified and receive the symptom and function profile for each patient. Thus, PCCs can review their patients’ symptoms and function to assist in clinical decision-making before initial treatment only if patients fill out the questionnaire online. The patients are surveyed again 12 months after the start of treatment (and also, if the PCCs so choose, additionally after 6 months and annually after 1 year). The follow-up survey is organized locally, i. e. centers send out questionnaires and reminders but are assisted by a centrally developed excel-based reminder tool to allow for uniform processes. The target recruitment rate for the centers is 75%.

### Linkage and transfer of data

Data are thus stored in two different environments, the PROM database that includes all PROMs, and the routinely data collected data in the center. At recruitment, patient identifiers are assigned to each participant with the consent form / the paper questionnaire / the online password. Patient identifiers together with the password allow accessing the questionnaire at baseline and follow-up. In case of paper questionnaire use, patient identifiers as part of the paper questionnaire are entered by the LDC into the PROM database. Either way, PCCs enter the patient identifiers into a software (“OncoBox Prostate”) that has been in use in PCCs before that and was originally meant to make plausibility tests for routine data. Within PCO it is also used to locally match routine data with PROM data using the patient identifier. Data are then pseudonymized in the OncoBox Prostate. To allow for the primary study purpose — comparing outcomes across providers — pseudonymized patient data (i.e., not identifiable for the receiver) are transferred to the LDC at least once a year. The matched dataset is transferred to the LDC via a secure file-sharing system. The following analyses are based on all data transferred by April 2018.

### Statistical analyses

We present a description of a study sample with absolute and relative frequencies of risk classification, definitive treatment, androgen deprivation therapy, and active surveillance /watchful waiting prior to recruitment, as well as comorbidities, nationality, health-insurance status, and education. Statistical analyses were carried out with R version 3.5.1. For patients recruited in 2017, comparisons with the center population are also presented for risk classification and definitive treatment. Differences in recruitment rates between centers are reported, both overall and relative to treatment, as well as variation in the use of online or paper questionnaires. Chi-squared tests were conducted to investigate differences in frequency distributions between groups.

## Results

Forty-four PCCs transferred data for 3094 patients who were recruited between July 2016 and March 2018, resulting in an overall response rate of 39.3% of the eligible patients, with wide variation between centers — ranging from 4.7% to 94.6% (not presented). The online option was used by 13.8% of patients. Seventy-seven percent of the patients in the sample were suffering from low-risk or intermediate-risk disease; 1.3% had advanced (N1) disease (Table 1). Of the patients sampled, 3.2% had received androgen deprivation therapy, 2.6% active surveillance, and 0.5% watchful waiting prior to recruitment. The patients’ mean age was 66 (range 39–85). Ninety-six percent of the patients had German nationality, 77% had statutory health insurance, and one-third of them had received a university entrance qualification certificate (high-school diploma). Sixty-three percent of the recruited patients had no reported comorbidities. Whereas the documentation was complete for risk classification, treatments, and age, there was a missing data rate of below 5% for patient-reported nationality, health-insurance status, and educational level. However, the missing data rate was over 20% for center-documented comorbidities, with 14 centers not documenting comorbidities at all.

**Table 1:** Characteristics of the patients included in the study (*n* = 3094)

Variable	n	%
Risk class ^a^		
Low, localized	521	16.8
Intermediate, localized	1399	45.2
High, localized	991	32.0
Locally advanced	144	4.7
Advanced (N1)	39	1.3
Data missing	0	
Therapy		
RPE alone	2608	84.3
RT alone	221	7.1
RPE + RT	172	5.6
AS/WW	79	2.6
RCP	10	0.3
Other local therapy	4	0.1
Data missing	0	
ADT prior to recruitment		
No	2992	96.8
Yes, finished	69	2.2
Yes, ongoing	33	1.0
Data missing	0	
AS prior to recruitment		
Yes	80	2.6
No	3014	97.4
Data missing	0	
WW prior to recruitment		
Yes	14	0.5
No	3080	99.5
Data missing	0	
Comorbidities		
0	1521	62.7
1–2	856	35.3
> 2	50	2.1
Data missing	667	
Nationality		
German (including multiple nationalities)	2840	96.3
Other	109	3.7
Data missing	145	
Health insurance		
Statutory	2275	77.2
Private	662	22.5
Other/none	11	0.4
Data missing	146	
School-leaving certificate		
Lower secondary school	1162	39.5
Intermediate secondary school (West)	603	20.5
Intermediate secondary school (East)	127	4.3
FHSR	368	12.5
University entrance certificate	607	20.6
Other	49	1.7
None	24	0.8
Data missing	154	
Age: mean (range) (data missing)	66.0 (39–85) (0)	

^a^Risk class in accordance with the German Guideline for Prostate Cancer [14]

*ADT* Androgen deprivation therapy; *AS* Active surveillance; *FHSR Fachhochschulreife,* entrance certificate for a higher technical college/university of applied science; *RCP* Radical cystoprostatectomy; *RPE* Radical prostatectomy; *RT* Radiotherapy; *WW* Watchful waiting

An examination of patients recruited in 2017 alone allows insight into how well the sample represents the population. In comparison with the center population, in which patients with low-risk, localized disease represented 21%, the latter only made up 17% of the study sample, whereas patients with intermediate-risk disease represented a slightly higher proportion of the study sample (45% compared to 41% in the center population) (Table 2). Ninety percent of the recruited patients received radical prostatectomy or radical prostatectomy and radiotherapy. Patients treated with radical prostatectomy were therefore overrepresented in the sample, and all other treatment groups were underrepresented (Table 2).

**Table 2:** Patient recruitment relative to risk classification and type of treatment

Population			Sample	
n	%		n	%
Risk classification				
1318	21.41	Localized — low risk	409	17.32
2498	40.58	Localized — intermediate risk	1052	44.54
1921	31.21	Localized — high risk	753	31.88
318	5.17	Locally advanced	116	4.91
100	1.63	Advanced	32	1.35
Type of treatment				
4153	67.53	Radical prostatectomy (RPE) ^a^	2121	89.80
45	0.70	Radical cystoprostatectomy (RCP) ^a^	7	0.30
1018	15.94	Radiotherapy (RT)	130	5.50
149	2.33	Brachytherapy (BT)	37	1.57
782	13.36	Active surveillance (AS)/watchful waiting (WW)	63	2.67
9	0.14	Other local therapy	4	0.17

N.B.: The data are restricted to primary patients in 2017 in centers that recruited throughout the year; ^a^ including radiotherapy following radical (cysto)prostatectomy; there was a significant difference between the population and sample with regard to the distribution of risk classification (χ^2^ = 21.80; *P* value = 0.00022) and to the type of definitive therapy (χ^2^ = 457,52; *P* value < 2.2 * 10^−16^)

Recruitment of patients relative to the different treatment options also varied widely between the centers, with medians of 51% and 6% for eligible patients treated with radical prostatectomy or radiotherapy and 0% of eligible patients treated with brachytherapy and active surveillance/watchful waiting (Supplementary Table 1).

## Discussion

Drawing conclusions from observational data requires thoughtful study design and evaluation of the data, as well as a sensitive interpretation of findings. A thorough discussion of the data collection process and potential pitfalls of the data are necessary in order to achieve this.

The data from clinical documentation (routine data) in this study were close to complete with the study benefitting from documentation that was established beforehand. The rate of missing information in patient-reported items was below 5%. Particular emphasis should be given to the poor recruitment of nonsurgically treated patients in many PCCs. This reflects decentralized structures in the centers and lack of cooperation between urologists and radiotherapists at various sites. Active surveillance/watchful waiting patients are similarly underrepresented in the study sample, reflecting the office-based treatment of these patients by practitioners outside the PCCs. This may also explain the underrepresentation of low-risk patients.

It should be noted that the PCCs were invited to participate on a voluntary basis, without any financial compensation. Starting with 24 PCCs in 2016, the number soon rose. Although the target value for recruitment was set at 75% of eligible patients, centers that did not reach this number were not excluded, and measures (such as workshops and monthly monitoring) were developed to improve recruitment. A thorough evaluation if these measures affect recruitment however is difficult to conduct. Efforts need to be made not only to reduce the enormous variation in recruitment rates across PCCs, but also to ensure that a representative proportion of patients who receive nonsurgical treatment options are included. In addition to the poor recruitment of nonsurgical patients, the collection of data on comorbidities (optional in the TNGR) emerged as a major challenge for the PCCs: 14 of the 44 data-transferring centers did not report comorbidities at all. The overall burden of comorbidities is lower than in most similar studies, although some other studies have reported an even lower comorbidity burden [1, 15–17]. However, this is suggestive of underreporting and with some centers not documenting comorbidities at all, two modifications to collect these may be considered for the future: First, reimbursement of centers for this additional data collection, and second, collecting comorbidities via the patient questionnaire. The first option would further increase costs. One strategy to generally keep costs low from the beginning was to collect PROM data online. Yet, less than 14% of patients used the online survey. One strength of the data collection infrastructure described here is that the patient population is known at each PCC, allowing recruitment rates to be calculated for each PCC and for different patient strata — which is rarely possible in comparable studies, whether observational or interventional. Although a recruitment rate of 39% may seem low in comparison with other studies, the rate reflects the true value of *all* eligible patients, in contrast to many other studies with undefined or unclear denominators. 70.4% of participants filled out a follow-up questionnaire (not reported).

The primary purpose of the PCO study and the TNGR is to enable health-care providers who are treating patients with prostate cancer to compare their own work with others and to initiate processes of mutual learning. Since quality improvement initiatives are collective efforts that benefit from other researchers’ experience, the authors would be grateful to receive comments on the issues discussed in this article.

## Supplementary information

**Additional file 1: Table 1.** Patient recruitment relative to center according to treatment (for patients recruited in 2017).

## Data Availability

The datasets generated during and/or analysed during the current study are not publicly available as per se patient consent form.

## Abbreviations

EPIC-26: Expanded prostate cancer index composite 26-item version
ICHOM: International consortium for health outcomes measurement
LDC: Local data center
PCC: Prostate cancer center
PCO study: Prostate cancer outcomes study
PROM: Patient-reported outcome measures
PSA: Prostate-specific antigen
TNGR: TrueNTH Global Registry

## Footnotes

1 The study protocol (in German and English) and ethics committee documents can be downloaded from the study website at https://www.pco-study.com/centersinfo.
