# Supplementary material for: A multicenter paper-based and web-based system for collecting patient-reported outcome measures in patients undergoing local treatment for prostate cancer: first experiences
Source: J Patient Rep Outcomes. 2020 Jul 13;4:56. doi: 10.1186/s41687-020-00224-7 (PMC7359206; doi:10.1186/s41687-020-00224-7)
Supplement: Supplementary file 1 — Additional file 1: Table 1. Patient recruitment relative to center according to treatment (for patients recruited in 2017). [file 41687_2020_224_MOESM1_ESM.docx]

**Supplementary Table 1**Patient recruitment relative to center according to treatment (for patients recruited in 2017)

|  | Overall | RPE | RT | BT | AS/WW |
| --- | --- | --- | --- | --- | --- |
|  | 2362 | 2121 | 130 | 37 | 63 |
| Mean for centers |  | 46.48 | 13.80 | 13.95 | 7.92 |
| Maximum |  | 100.00 | 83.33 | 83.78 | 57.89 |
| 75% quartile |  | 96.23 | 15.63 | 16.67 | 5.20 |
| Median |  | 50.79 | 6.41 | 0.00 | 0.00 |
| 25% quartile |  | 24.13 | 0.00 | 0.00 | 0.00 |
| Minimum |  | 0.00 | 0.00 | 0.00 | 0.00 |

N.B.: The data were restricted to primary patients in 2017; 11 patients with other local therapy or cystoprostatectomy were excluded.

AS, active surveillance; RPE, radical prostatectomy; RT, radiotherapy; WW, watchful waiting.
